# Mass Separation by Metamaterials

**DOI:** 10.1038/srep21971

**Published:** 2016-02-25

**Authors:** Juan Manuel Restrepo-Flórez, Martin Maldovan

**Affiliations:** 1 School of Chemical & Biomolecular Engineering, Georgia Institute of Technology, Atlanta, Georgia 30332,USA; 2 School of Physics, Georgia Institute of Technology, Atlanta, Georgia 30332, USA

## Abstract

Being able to manipulate mass flow is critically important in a variety of physical processes in chemical and biomolecular science. For example, separation and catalytic systems, which requires precise control of mass diffusion, are crucial in the manufacturing of chemicals, crystal growth of semiconductors, waste recovery of biological solutes or chemicals, and production of artificial kidneys. Coordinate transformations and metamaterials are powerful methods to achieve precise manipulation of molecular diffusion. Here, we introduce a novel approach to obtain mass separation based on metamaterials that can sort chemical and biomolecular species by cloaking one compound while concentrating the other. A design strategy to realize such metamaterial using homogeneous isotropic materials is proposed. We present a practical case where a mixture of oxygen and nitrogen is manipulated using a metamaterial that cloaks nitrogen and concentrates oxygen. This work lays the foundation for molecular mass separation in biophysical and chemical systems through metamaterial devices.

Rational design of metamaterial structures enables unprecedented manipulation of physical properties in research areas that include optics, acoustics, mechanics, and thermal transport^1^,^2^. Initially, metamaterial cloaking shells designed by coordinate transformations and conformal mapping were shown to render objects invisible to light^3^,^4^,^5^,^6^. In recent years, metamaterial theory has expanded beyond the concept of invisibility, making possible a variety of novel optical devices such as light concentrators, splitters, and perfect lenses^4^. The principle behind optical metamaterials is the invariance of Maxwell’s equations under coordinate transformations. Following the remarkable success in optics, metamaterials have been further extended to other research areas by recognizing that the fundamental equations of sound, elastic vibrations, matter-wave phenomena, as well as those of heat (and mass transfer under steady-state conditions), are also invariant under coordinate transformations^7^,^8^,^9^,^10^,^11^. This extension of metamaterial theory and concepts has led to a large number of counter-intuitive methods to control optical, acoustical, mechanical, and thermal properties^7^,^8^,^9^,^10^,^11^.

Mass flow manipulation is of utmost importance in many physical processes in chemical and biomolecular science, since separation and catalysis require precise control of the diffusion of relevant species^12^. Separation systems are critically important in several different research areas ranging from chemical manufacturing, recovery of biological solutes from wastes, to semiconductor crystal growth, and generation of artificial kidneys. A systematic method to achieve precise manipulation of molecular diffusion paths is provided by coordinate transformations and metamaterials. Despite such fundamentally new opportunities to control mass diffusion, to date, mass diffusion metamaterials have been largely unexplored^9^,^13^,^14^,^15^.

Techniques to control *heat* diffusion in ways that yield transparent devices were initiated with the design of neutral inclusions back in the 50’s^16^,^17^. More recently, the invariance of diffusion equations was studied at steady-state by Milton *et al.*^7^; and results were applied to the design of *heat cloaks* by Chen *et al.*^18^ and Fan *et al.*^19^. However, a complete description of the problem, including transient regime and heat sources, was introduced by Guenneau *et al.*^8^. Thermal metamaterials were subsequently extended to the design of thermal rotators and concentrators^20^,^21^,^22^ and the experimental demonstration of these devices shows that metamaterials can be practically applied to manipulate heat transfer^20^,^23^,^24^,^25^,^26^,^27^,^28^,^29^. More recently, the design of metamaterials with multi-physics range of action has also been introduced where structures control thermal and electrical fields^30^,^31^,^32^.

Molecular mass diffusion can be described by similar equations to those governing heat flux. However, there are two important differences between heat and mass diffusion processes. First, mass diffusion is commonly found in practical applications as a multi-component problem, in the sense that more than one species are being transported. Second, when dealing with multiphases, one has to consider the possibility of a discontinuity in the concentration at interfaces due to different compound solubilities in the phases. In a more general case, the chemical potential gradient rather than the concentration gradient governs molecular transport and a generalized Fick’s law must be considered^33^. To use the standard Fick’s law, one must therefore ensure that the solubility of the compounds does not significantly vary through the system.

In this work, we introduce a novel physical approach for mass separation based on mass-diffusion metamaterials that can cloak one compound while concentrating the other, being this the first metamaterial that can sort chemical and biomolecular species. Such novel metamaterial provides the basis for a new method to manipulate mass diffusion and achieve separations in biomedical, biophysics, and chemical applications. We employ coordinate transformations to the Fick’s law equation to simultaneously manipulate the diffusion paths of different species in both transient and steady state regimes. The design of an ideal, non-homogeneous, anisotropic mass-separator metamaterial device is first introduced. We next present a design strategy that allows for experimental realization of such metamaterial using homogeneous isotropic materials. A practical proof-of-concept is introduced by separating a binary mixture of oxygen and nitrogen diffusing through a polymeric matrix. The simultaneous metamaterial manipulation of two different chemical species has not been reported in the literature. This work paves the way to achieve mass separations using metamaterials devices in chemical and biomolecular science and technology.

## Results

Let us consider an arbitrary binary dilute mixture consisting of species A and B diffusing in a background medium. Since, in a dilute solution, diffusion of A is independent of diffusion of B, we propose a metamaterial structure that acts as a cloak for compound A and simultaneously as a concentrator for compound B, enabling the first demonstration of mass separation by metamaterials. The key concept in the design of such metamaterial structure is that the cloaking effects on A will not have a significant impact on the diffusion of B, while the concentrating effects on B will not have a significant influence on A. We thus perform two different coordinate transformations in the same spatial domain − each related to one of the compounds of interest (see Methods). To obtain mass separation, the two diffusion problems (cloaking and concentration) are initially treated independently in order to subsequently achieve them simultaneously.

**Figure 1:**
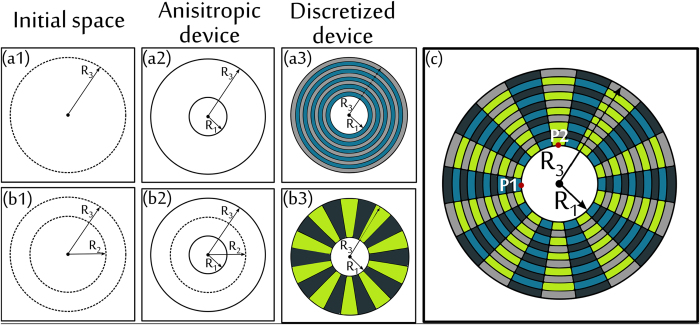
Mass separation metamaterials by coordinate transformations. (**a**) Cloaking: A point is transformed into a circle of radius *R*_1_ while the adjacent region is being compressed. Experimental realization of such transformation is obtained by a multilayer structure made of concentric rings. (**b**) Concentrator: Compression of a circular region 0 < *r* < *R*_2_ into 0 < *r* < *R*_1_ followed by expansion of the adjacent annulus from *R*_2_ to *R*_1_. The experimental realization is obtained by a lamellar structure. (**c**) Bi-functional multilayer device: the design requires four different materials depicted by dark blue, grey, light blue, and light green colors, respectively. P_1_ and P_2_ are selected points where concentrations of A and B are evaluated.

**Figure 2:**
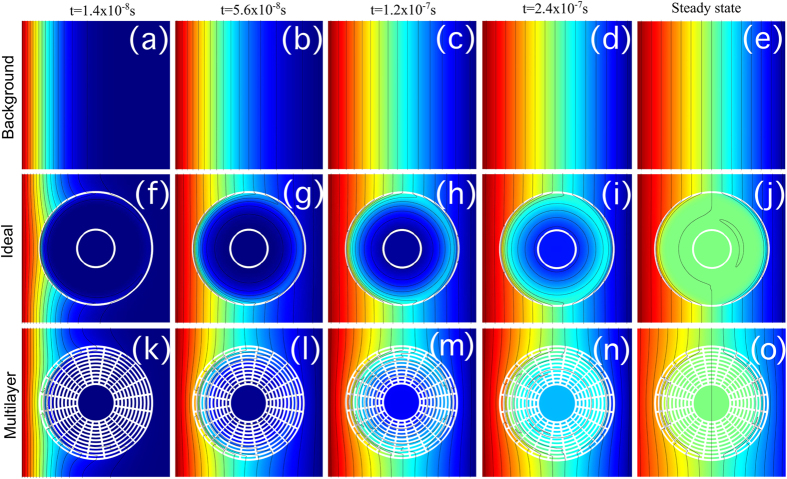
Cloaking of species A. Concentration profiles for *cloaked* compound A at different times and steady-state. Insets (**a–e**), background media without metamaterial. (**f–j**) Anisotropic homogeneous cloak. (**k–o**) Multilayer cloaking device.

**Figure 3:**
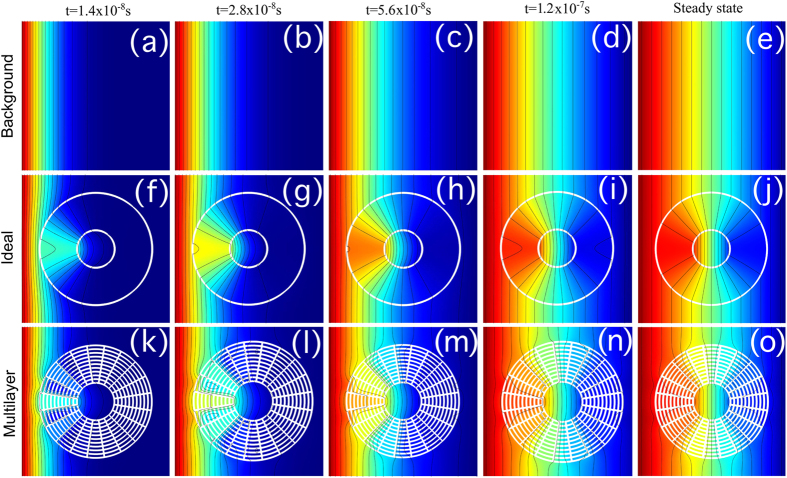
Concentration of species B. Concentration profiles for *concentrated* compound B at different times and steady-state. Insets (**a–e**), background media without metamaterial. (**f–j**) Anisotropic homogeneous concentrator. (**k–o**) Multilayer concentrator device.

**Figure 4:**
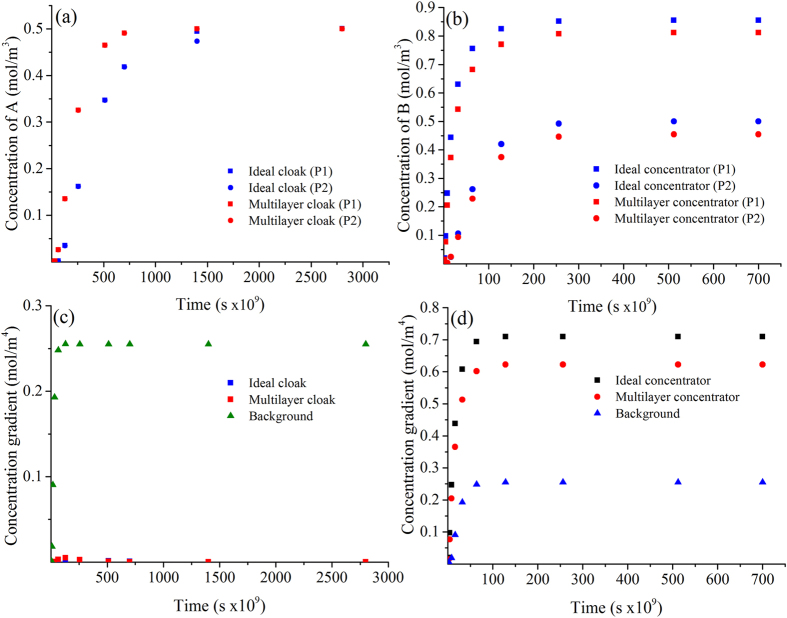
Time-dependent concentration (**a,b**) Concentration values at two different locations inside the mass-separator device (locations are highlighted in Fig. 1c with numbers 1 and 2) as a function of time for cloaked and concentrated species, respectively. (**c,d**) Concentration gradients inside the bi-functional device for cloaked and concentrated species.

As an initial proof-of-concept, we first introduce a metamaterial device for an arbitrary binary mixture diffusing in an inert media, which is capable of cloaking A while concentrating B (Fig. 1). The relative radial diffusivities are considered to be 0.1 and 10 for the cloaked and concentrated species respectively. The azimuthal diffusivities are calculated using equation (7) (see Methods). The spatial domain consists of a square of side 0.8 mm and the external and internal cloaking shell radius are *R*_3_ = 0.3 mm and *R*_1_ = 0.1 mm respectively (Figs 2 and 3). We assume that the solubility of compounds A and B is constant through the system. Results for the unperturbed concentration gradients (Figs 2 and 3a–e) and the concentration profiles of species A (cloaked) and species B (concentrated) are shown in Figs 2 and 3f–j, respectively. The metamaterial device works in both transient and steady-state regime for the relevant variables in the present application. A near zero concentration gradient for the cloaked compound (species A) is observed inside the device all along the process (Fig. 4c). On the other hand, a larger concentration gradient is observed for the concentrated compound (species B) (Fig. 4d). Comparison between concentration fields in the presence (Figs 2 and 3f–j) and absence (Figs 2 and 3a–e) of the metamaterial reveals that for both (cloaked and concentrated) species the field outside the device remains largely undisturbed (Figs 2 and 3g–j) except at early stages (Figs 2 and 3f).

Although these results provide the basis for mass separation by metamaterials, the requirements in terms of diffusion-coefficient anisotropy are difficult to fulfill experimentally by considering an homogeneous material. We thus propose a simple scheme that provides the prescribed anisotropy for simultaneous mass cloaking (A) and concentration (B) by using four isotropic materials. It is important to note that the solubility of (A) and (B) in the materials should be constant. A stack of layered materials with different diffusivities creates a system of resistors in parallel (longitudinal direction) and in series (perpendicular direction) in which the spatial anisotropy can be controlled^20^,^34^. For a *cloak*, resistors are required in parallel along the azimuthal direction and in series along the radial direction. A structure made of concentric rings with alternating diffusivities, as shown in Fig. 1a, satisfies such requirement. Contrarily, for a *concentrator*, resistors are in parallel along the radial direction and in series along the azimuthal direction. A lamellar structure as shown in Fig. 1b is thus required. Note the contrary requirements for a cloak or a concentrator in terms of the alignment of the layered materials. Effective medium approaches show that the radial and azimuthal diffusivities for bi-layered materials are given by 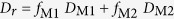, and 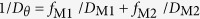 respectively, where *D*_M1_ and *D*_M2_ are the diffusivities of the homogeneous isotropic materials and *f*_M1_ and *f*_M2_ are the volume fractions. Note that although the same equations apply, radial and azimuthal diffusivities must be flipped in a concentrator with respect to a cloak.

We apply the discretization procedure described above to the diffusivity tensors of compounds A and B. In doing so, one must consider four constrains imposed by the effective medium solutions for cloaked and concentrated species. The minimum number of isotropic materials that will satisfy these constrains is four. One arrangement that yields the desired results is presented in Fig. 1c. Note that this metamaterial structure consists of simultaneous concentric rings and adjacent lamellae. The idea behind the design of such metamaterial is that the diffusivity for the cloaked compound is constant in the azimuthal direction, and for the concentrated species is constant in the radial direction. For simulations, 10 concentric layers were used in the radial direction and 20 layers in the azimuthal direction. The relative diffusivities of the homogeneous materials were calculated by the effective medium approach using radial and azimuthal diffusivities *D*_*r*_ and *D*_*θ*_ determined in the previous section. Without losing generality, we consider volume fractions *f*_M1_ = *f*_M2_ = 0.5. The material diffusivities are *D*_A,1_ = *D*_A,2_ = 19.95, *D*_A,3_ = *D*_A,4_ = 0.05, *D*_B,1_ = *D*_B,3_ = 19.95, *D*_B,2_ = *D*_B,4_ = 0.05. The concentration profiles for species A and B are shown in Figs 2 and 3k–o. From the plots, it is clear that both cloaking and concentrating effects for A and B respectively are simultaneously achieved. Some small disturbances in the iso-concentration lines are observed as a consequence of the discretization procedure. However, the performance of the multilayer device can be improved by increasing the number of layers. Figures 2 and 3 show that it is thus possible to construct a bi-functional metamaterial device that simultaneously controls the diffusion paths of two different species, cloaking A while focusing B, using *homogeneous isotropic* materials.

Time-dependent concentrations for species A and B at two different locations (points 1 and 2 in Fig. 1) are shown in Fig. 4a,b for both ideal and multilayer designs. Three conclusions can be stated: first, the performance of the ideal device is comparable to that of the multilayer device for both cloaked and concentrated species; however, when comparing ideal and multilayer devices there are slight differences in the concentrations at the beginning of the process and the multilayer cloak reaches the steady-state regime faster than the ideal device. Second, when comparing concentrations at the two different locations, it is clear that for the cloaked compound A the concentration is constant. On the other hand, for the concentrated compound B, it is observed, at steady-state, a concentration difference of around 80% between the two points evaluated. Finally, the steady-state regime is reached faster in the case of the concentrated species than in the case of the cloaked species. If we compare the characteristic times to reach steady-state in the ideal device, for the cloaked compound (species A) the time required is ~1.4 × 10^−6^ s while for the concentrated species B is only ~2.56 × 10^−7^ s. Figure 4c,d show the time-dependence of the concentration gradient at the core of the metamaterial device for cloaked and concentrated species, from which the transient performance can be evaluated. For the cloaked compound (Fig. 4c), the internal concentration gradient − for ideal and multilayer cases − is approximately zero during the entire experiment. In addition, in the case of the concentrated species (Fig. 4d), the concentration gradient in the device is always greater than that in the background media (without metamaterial). Importantly, the differences in concentration for compounds A and B can be employed for the design of metamaterial devices with applications in chemical and biophysical separations. The design presented achieves separation of compounds A and B, since on the left hand side of the core we have (C_B_ > C_A_), and on the right (C_B_ < C_A_). In one practical application, the core can consist of two semi-circular cylinders, after steady-state, the core can be extracted and compounds A and B are separated in the two cylinders (one with majority of A and the other with B).

We note that replacing the structure by a single material is less efficient for separation. Using a single material, the optimal field distribution to obtain separation in the core at steady-state will be having a zero gradient for A and the external gradient for B (or viceversa). Even such a configuration is less efficient than our design. In our results, the concentration gradient for B in the core is larger than the external gradient due to concentration effects, providing larger separation efficiency. This difference in efficiency arises from the fact that a single material cannot shield A and concentrate B at the same time. In addition, if we consider the transient regime, a layered structure can be better to protect a region from a diffusing compound than a single material^23^. Although heat and mass diffusion can be described by similar equations, it should be noted that simultaneous manipulation of two or more species is only possible in mass diffusion. This kind of treatment is different from previous work in heat transport where cloaking or concentrating shells have been reported. Note that these two effects cannot be achieved simultaneously in the case of heat.

**Figure 5:**
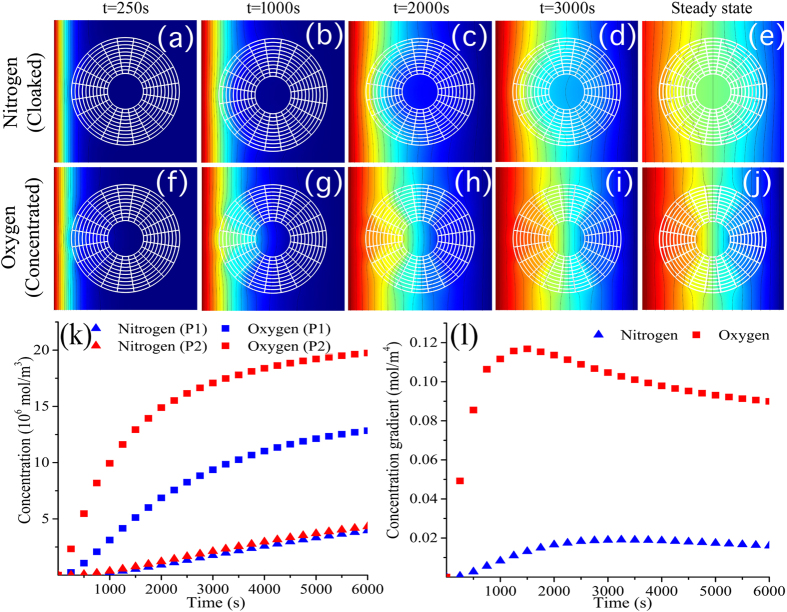
Mass separation of oxygen and nitrogen. (**a–e**) Cloaking of N_2_ and (**f–j**) concentration of O_2_ at different times and steady-state. The simultaneously manipulation of different species enables a practical approach for mass-separation of mixtures. (**k**) Oxygen and nitrogen concentration at two different locations inside the device. (**l**) Concentration gradients for cloaked N_2_ and concentrated O_2_ compounds.

We next apply the proposed concept of a mass-separator metamaterial device to a binary mixture of oxygen and nitrogen. The mixture is considered to be diffusing through a polymer block of Butadiene acrylonitrile (68/32). We designed a metamaterial device that simultaneously cloaks nitrogen and concentrates oxygen. We use the scheme introduced in the previous section and selected four homogeneous isotropic materials (Natural rubber, kraton KG VTEOS, Methyl rubber and Hydrin 100 with filler) based on the approximate fulfillment of two criteria: the respective solubility of oxygen and nitrogen is the same as in the background media, the diffusivities of oxygen and nitrogen are similar to those previously calculated using equation (7) in the Methods Section. The properties of the constituent materials are given in Table 1^35^. The concentration profiles for O_2_ (concentrated) and N_2_ (cloaked) are presented in Fig. 5 for the first 3000 seconds and steady-state. Figure 5 shows how the effects of the mass-separator device can be verified for the two species (O_2_ and N_2_), as the N_2_ molecules are directed around the core while the O_2_ are directed towards the core. Some disturbances of the field outside the device can be observed. However, in mass-separation applications, the manipulation of internal diffusion paths rather than unaltered fields outside the device is relevant. Figure 5k,l show the concentration of oxygen and nitrogen at the two different locations in the metamaterial device and the concentration gradients within the core. Note that the concentration of nitrogen is always lower than the concentration of oxygen, demonstrating the possible practical applications of metamaterials in mass separation.[Table t1]

In conclusion, we introduced a novel approach to rationally design mass-separator metamaterial devices that can cloak one molecular species while concentrate another, enabling the development of the first metamaterial for sorting molecular compounds. We proved that there exists a feasible geometric metamaterial configuration to achieve such effects using isotropic homogeneous materials. It is important to note that the proposed approach for mass separation is based on transforming the standard Fick’s law equation for diffusion and therefore considers similar material solubilities. More generally, mass concentration can be discontinuous at interfaces due to the different solubilities. A more general theoretical approach should include the chemical potential as the variable in the transformation, since the chemical potential rather than the concentration gradient is the driving force in general diffusion processes. Future work will focus on designing mass-separator metamaterials made of constituent materials with different solubilities. The simultaneous control of the diffusion of chemical species is extremely relevant in biophysics, biomolecular, and chemical science and technology, both in separation and catalysis processes. The concept of mass separation by metamaterials introduced here thus provides a novel tool to control the diffusion paths of chemical and biomolecular species, opening the opportunity to unprecedented simultaneous manipulation of different molecular species.

## Methods

We describe the diffusion of a dilute gas mixture in an isotropic background media by Fick’s law (equation 1), where *C*_*i*_ is the concentration of species *i* and *D*_*i*_ is the diffusivity in the background material.


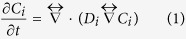


The new equation in the transformed space^9^ is given by equation (2)


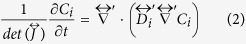


where the transformed diffusivity tensor 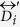 is given by,


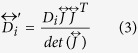


where 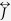 is the Jacobian of the transformation matrix. The equation is invariant in steady-state, but it is not invariant in unsteady state, since there is no physical parameter on the left-hand side to absorb the determinant. The steady-state or the reduced version of the equation can be used to design cloaks and concentrators.

A linear transformation^5^ is used in the design of both cloaks and concentrators (equation 4), where *a* and *b* are constants^5^,^8^. For cloaking, the coordinate transformation consists of mapping a circle of radius *R*_3_ into an annulus bounded by circumferences of radius *R*_1_ and *R*_3_ (Fig. 1a). For a concentrator, two simultaneous operations are required, i.e. the compression of a circular region of radius *R*_2_ into a circular region of radius *R*_1_ followed by the expansion of the region *R*_2_ < *r* < *R*_3_ (Fig. 1b). The radial (*r*) and azimuthal (θ) relative diffusivities for a cloak (*cl*) and for a concentrator (*cn*) are given by equations (5) and (6), respectively. In both cases, the product of the radial and azimuthal relative diffusivities is one (equation 7). This identity can be used to design cloaks and concentrators by considering the inverse of the radial or azimuthal diffusivities being constant throughout the device (equation 7)^22^,^36^. If *C* > 1, a concentrator is obtained, while for *C* < 1, a cloaking device is obtained^22^. The key advantage of this design strategy is that allows to achieve cloaks and concentrators with simple parameters (e.g. homogeneous, finite constant diffusivity)^22^,^36^.


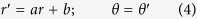



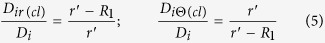



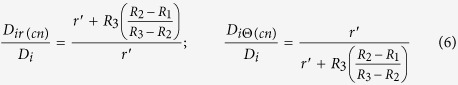



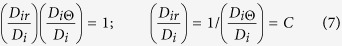


## Additional Information

**How to cite this article**: Restrepo-Flórez, J. M. and Maldovan, M. Mass Separation by Metamaterials. *Sci. Rep.* **6**, 21971; doi: 10.1038/srep21971 (2016).

## Figures and Tables

**Table 1 t1:** Material properties^35^.

Material	D_N2_ (m/s)	S_N2_(mol/m^3^Pa)	D_O2_ (m/s)	S_O2_(mol/m^3^Pa)
Background: Butadiene/Acrylonitrile (68/32)	1.5 × 10^−11^	1.3 × 10^−5^	2.8 × 10^−11^	2.8 × 10^−5^
M1: Natural rubber	1.17 × 10^−10^	2.2 × 10^−5^	1.6 × 10^−10^	4.9 × 10^−5^
M2: kraton KG VTEOS	1.14 × 10^−10^	2.9 × 10^−5^	1.4 × 10^−11^	6.1 × 10^−4^
M3: Methyl rubber	7.9 × 10^−12^	2.0 × 10^−5^	1.7 × 10^−10^	4.1 × 10^−6^
M4: Hydrin 100 with filler	8 × 10^−13^	6.4 × 10^−5^	3.1 × 10^−12^	2.3 × 10^−5^
